# Rhesus macaque *IFITM3* gene polymorphisms and SIV infection

**DOI:** 10.1371/journal.pone.0172847

**Published:** 2017-03-03

**Authors:** Michael Winkler, Sabine Gärtner, Florian Wrensch, Michael Krawczak, Ulrike Sauermann, Stefan Pöhlmann

**Affiliations:** 1 Infection Biology Unit, Deutsches Primatenzentrum, Leibniz Institute for Primate Research, Göttingen, Germany; 2 Institute of Medical Informatics and Statistics, Christian-Albrechts University, Kiel, Germany; 3 Infection Models Unit, Deutsches Primatenzentrum, Leibniz Institute for Primate Research, Göttingen, Germany; Emory University School of Medicine, UNITED STATES

## Abstract

Interferon-induced transmembrane proteins (IFITMs) have been recognized as important antiviral effectors of the innate immune system, both in cell culture and in infected humans. In particular, polymorphisms of the human *IFITM3* gene have been shown to affect disease severity and progression in influenza A virus (FLUAV) and human immunodeficiency virus (HIV) infection, respectively. Rhesus macaques (*Macaca mulatta*) are commonly used to model human infections and the experimental inoculation of these animals with simian immunodeficiency virus (SIV) is one of the best models for HIV/AIDS in humans. However, information on the role of IFITM3 in SIV infection of rhesus macaques is currently lacking. We show that rhesus macaque (rh) IFITM3 inhibits SIV and FLUAV entry in cell culture, although with moderately reduced efficiency as compared to its human counterpart. We further report the identification of 16 polymorphisms in the *rhIFITM3* gene, three of which were exonic and synonymous while the remainder was located in non-coding regions. Employing previously characterized samples from two cohorts of SIV-infected rhesus macaques, we investigated the relationship between these *rhIFITM3* polymorphisms and both AIDS-free survival time and virus load. In cohort 1, several intronic polymorphisms were significantly associated with virus load or survival. However, an association with both parameters was not observed and significance was lost in most cases when animals were stratified for the presence of MHC allele Mamu-A1*001. Moreover, no significant genotype-phenotype associations were detected in cohort 2. These results suggest that, although IFITM3 can inhibit SIV infection in cell culture, genetic variation in *rhIFITM3* might have only a minor impact on the course of SIV infection in experimentally infected animals.

## Introduction

Interferon-induced transmembrane proteins are encoded by a gene family of diverse function in vertebrates [1–3]. A subgroup of immunity-related IFITMs (IFITM1-3) has been recognized recently to inhibit multiplication of a broad range of viruses including, but not limited to, influenza A viruses (FLUAV), flaviviruses, coronaviruses, filoviruses and lentiviruses [4–11]. IFITM proteins are type II transmembrane proteins and consist of a variable cytoplasmic N-terminus followed by a conserved intramembrane domain, an intracellular loop, a transmembrane domain and a short variable and luminal C-terminus [12–14]. It is well established that IFITMs inhibit viral entry [11]. While the exact mechanism still has to be elucidated, IFITMs seem to act through the arrest of virus-host cell membrane fusion at the hemifusion step by altering membrane fluidity [15, 16]. Despite their broad antiviral activity, IFITMs can also promote entry or assembly of selected viruses [17, 18], indicating that they belong to a pathway that can positively and negatively regulate viral infection.

All immunity-related IFITMs (IFITM1-3) have been shown to affect early stages of human and simian immunodeficiency virus (HIV, SIV) replication, with IFITM2 and IFITM3 inhibiting virus entry [8, 19, 20]. In addition, IFITMs may target later stages of the viral replication cycle such as Gag expression and proteolytic processing of Env [8, 21, 22]. The antiviral activity of IFITM1 can restrict HIV-1 cell-to-cell spread, and mutations in *env* and *vpu* have been identified that rescue the virus from inhibition by IFITM1 at the cost of reduced viral fitness [21, 23]. Moreover, IFITMs are incorporated into lentiviral particles thereby reducing viral infectivity even though inhibition by IFITMs and virion incorporation of IFITMs were not found to be intimately related [21, 24, 25]. Finally, unlike other antiviral effector proteins of innate immunity, IFITMs do not seem to be antagonized by viral factors [22].

Different lines of evidence suggest that IFITMs can limit viral spread and reduce disease burden in the infected host. Thus, absence of IFITM3 expression was found to be associated with reduced FLUAV spread and pathogenesis in experimentally inoculated mice [26, 27]. Moreover, a polymorphism in the human *IFITM3* gene (rs12252 T>C) was found in several studies to be associated with severe influenza [16, 27–29], although one study reported an association with mild influenza only [30]. The rs12252 T>C polymorphism is believed to alter *IFITM3* mRNA splicing [27] and to result in the expression of an IFITM3 variant lacking the first 21 amino acids, which displays reduced antiviral activity against FLUAV upon directed expression in cell culture [31]. Conversely, the truncated protein exerts increased anti-HIV-1 activity in transfected cells, due to preferential localization at the cell surface [32], the site of HIV-1 entry and exit from target cells. However, an association between rs12252-C and more rapid progression to AIDS, but not risk of HIV-1 infection, has been demonstrated [33], and the reason for the discrepancy between these results and the increased anti-HIV-1 activity of truncated IFITM3 in cell culture is at present unclear. Finally, a recent study demonstrated that HIV-1 variants sexually transmitted between individuals are resistant against blockade by IFITM proteins indicative of a strong selection pressure exerted by IFITMs [34, 35]. Moreover, IFITM expression was shown to be a major contributor to the blockade of HIV-1 by treatment of cells with IFN [34], suggesting that IFITMs constitute important defenses against HIV-1 infection.

Non-human primates are an important animal model for infectious diseases in humans. In particular, experimental SIV infection of rhesus macaques has contributed substantially to our current understanding of HIV/AIDS. Polymorphisms in several immune-relevant gene loci such as *MHC* or *KIR* are associated with the transmission and course of disease in SIV infected rhesus macaques and HIV-1 infected humans [36, 37]. Some primate *IFITM* gene products were recently shown to have antiviral activity against SIV and HIV-1 [19, 20]. However, these analyses did not include rhesus macaque so that it is presently unknown whether rhesus macaque (rh) IFITM3 also inhibits SIV infection in this species. Moreover, no *rhIFITM3* gene polymorphisms have been described so far or their modulatory effect upon infection by SIV or other viruses studied. Therefore, we systematically searched for polymorphisms in *rhIFITM3* and assessed their association with viral load at set point and disease progression in the context of SIV infection.

## Materials and methods

### Cells and plasmids

Human embryonal kidney (HEK) 293T cells (ATCC CRL-3216) were cultivated in Dulbeccos Modified Eagles Medium (DMEM), supplemented with 10% fetal calf serum (FCS), L-glutamine and penicillin/streptomycin in a humidified atmosphere containing 5% CO_2_. The cells were obtained from collaborators and their identity was verified by short tandem repeat (STR) analysis. Plasmids encoding Machupo virus glycoprotein (MACV-GPC), murine leukemia virus envelope protein (MLV-Env), FLUAV hemagglutinin (HA) and neuraminidase (NA), vesicular stomatitis virus glycoprotein (VSV-G) and SIV envelope protein (SIV-Env) were previously described [6, 7]. The MLV-based vector pQCXIP (Clontech, Heidelberg, Germany) encoding human and rhesus macaque IFITM3 proteins, chloramphenicol-acteyltransferase (CAT) or firefly luciferase as well as the MLV gag-pol encoding plasmid were also reported before [6, 7].

### Production of MLV particles

For the production of MLV particles encoding IFITM proteins, we followed a published protocol [6, 7]. In brief, 293T cells were cotransfected with vector pQCXIP harboring *rhIFITM3*, *huIFITM3* or *CAT*, an MLV gag-pol-encoding plasmid and a plasmid encoding vesicular stomatitis virus glycoprotein (VSV-G). For the production of reporter particles required for the analysis of IFITM3-mediated inhibition of viral entry, 293T cells were cotransfected with vector pQCXIP encoding firefly luciferase, an MLV gag-pol encoding plasmid and a plasmid encoding the viral glycoprotein to be tested. Medium was changed at 8 h and supernatants were harvested at 48 h post transfection, passed through a 0.45 μm filter, aliquoted and stored at -80°C.

### Analysis of the antiviral activity of rhesus macaque IFITM3

The antiviral activity of rhIFITM3 was analyzed as described previously [6]. In brief, 293T cells seeded at 10^4^ cells per well in a 96-well plate were spin-oculated [38] at 4,000×g for 30 minutes with IFITM3 or CAT encoding vectors. After incubation for 48h at 37°C, the medium was replaced by 50μl of fresh culture medium followed by the addition of 50μl of MLV vectors harboring the viral glycoproteins to be analyzed and normalized for equal luciferase activity upon transduction of control cells. After incubation for 8h, medium was replaced by fresh culture medium and luciferase activities in cell lysates were analyzed at 72h post infection, employing a microplate reader, Plate CHAMELEON V (Hidex, Turku, Finland), and a commercial luciferase assay system (Promega, Mannheim, Germany) or Beetle-Juice (PJK, Kleinblittersdorf, Germany) kits.

### Animals

Genetic and phenotypic data from two cohorts of SIV-infected rhesus macaques (*Macaca mulatta*) of Indian origin, comprising a total of 94 individuals (19 female, 75 male), were screened retrospectively for *rhIFITM3* polymorphisms (Table 1). Samples of *MHC*-typed animals were selected that were infected intravenously, intrarectally or via tonsils through a single dose of SIVmac239, SIVmac251 or SIVmac239/32H and which displayed the whole spectrum of disease progression, from rapid progression to long-term non progression, for each combination of virus and infection route. All macaques were either bred at the German Primate Centre and had known ancestry, or were from breeders in France, United Kingdom or the United States. The definition of early AIDS-defining illness was based on clinical, necropsy and histopathological findings as described [39]. The 39 animals included in the first cohort were previously used as a ‘screening cohort’ to search for polymorphisms influencing AIDS-free survival time and viral load in SIV infection [39]. It should be noted that no viral load data were available for some of the animals included in the survival analysis. The second cohort of 55 animals consisted of macaques more recently infected, and viral load data were available for all animals.

**Table 1:** Description of the cohorts.

	Cohort 1	Cohort 2
**No of macaques**	39	55
**Sex (%)**		
Male	28 (72)	47 (85)
Female	11 (28)	8 (15)
**Infecting SIVmac (%)**		
251	9 (23)	17 (31)
251/32H	13 (33)	0
251/32H/ex vivo, spleen	14 (36)	0
239	3 (8)	38 (69)
**Route of infection (%)**		
Intravenous	25 (64)	25 (45)
Intrarectal	4 (10)	13 (24)
Tonsillar	10 (26)	17 (31)
**Median age (year) at infection (range)**	4.3 (2–9)	4.4 (3.4–9)
**Mean survival (weeks) after infection (range)**	132.5 (6–901)	168 (22–591)
**Death with AIDS-related symptoms (%)**	25 (64)	41 (75)
Mamu-A1*001 positive (%)	22 (40)	11 (28)

### Ethics statement

The animals were housed and treated at the German Primate Centre under standard conditions which are in accordance with the German Animal Protection Act and the European Union guidelines on the use of non-human primates for biomedical research as described [40]. Briefly, all experiments were approved by an external ethics committee authorized by the Lower Saxony State Office for Consumer Protection and Food Safety (project licenses: 509.42502/08-02.95, 509.42502/08-13.98, 504.42502/08-03.90 (V+Ä), 33.9-42502-04-12/0820, 509-42502/08-04.03, 33.9.42502/04/017/07, 33.9.42502/04/72/08). According to §11 of the German Animal Welfare Act, the DPZ is permitted to breed and house non-human primates under license number 392001/7 issued by the local veterinary authorities. All macaques were under daily surveillance by veterinarians and animal caretakers. During quarantine, the animals were usually housed in groups of 2–3 animals per cage, with a minimum enclosure height of 180 cm and a cage volume of 3 to 4.5 m^3^. Animals in experiments had to be housed singly (cage dimensions [in cm]: 190h × 90w × 90d), but with constant visual, olfactory and acoustic contact to their roommates. Procedures for animal welfare and to minimize discomfort and suffering were undertaken in accordance with the recommendations of the Weatherall report “The use of nonhuman primates in research”. Monkeys were fed with dry monkey biscuits supplemented with fresh fruit or vegetables twice daily and fresh water access ad libitum. For environmental enrichment, animals were offered feeding puzzles, varying toys and wood sticks for gnawing. In addition, music was played. For DNA preparation, blood samples were drawn under anesthesia with 10 mg ketamine i.m. per kg body weight. Animals were humanely euthanized by an overdose of Pentobarbital-Natrium (Narcoren^®^, Merial, Hallbergmoos, Germany) under anesthesia either for experimental reasons without exhibiting clinical symptoms or in cases of suffering predefined by a scoring system of termination criteria that was approved by the external ethics committee and corresponds to the IACUC endpoint guidelines as described [40]. For the purpose of this study, no monkey was sacrificed since archival samples were used.

### DNA sequence analysis of *rhIFITM3*

The *rhIFITM3* gene was amplified from genomic DNA by PCR and screened for polymorphisms by re-sequencing. Primers for amplification were based on the *Macaca mulatta* chromosome 14 scaffold (Mmul_051212) [41]. We used this scaffold before to successfully amplify cDNAs of *rhIFITM* genes [6]. Amplification of genomic *IFITM3* sequences (LOC697829) was performed using primers mamuIFITM3gen5-1forA (5’-TTTGTTCCGCCCTCATCTGG-3’) and mamuIFITM3gen5-1rev (5’-TCTGAGATCCACGCTCAGGA-3’). Primers were designed using primer-BLAST [42] to specifically avoid amplification of *rhIFITM3(2)* (LOC697564) or *rhIFITM3* pseudogenes. Reaction mixtures contained 100 ng genomic DNA, 50 pmol of each primer, 100μM dNTP mix, 10% betain, 1x HF synthesis buffer and 1 U Phusion DNA polymerase. PCR cycling started with an initial denaturation step at 95°C for 5 min, followed by 40 cycles with denaturation at 95°C for 30 s, annealing at 68°C for 30s and elongation at 72°C for 1:40 min followed by a post amplification step at 72°C for 10 min. PCR products were gel purified and sequenced using ABI Big Dye chemistry and primers mamuIFITM3gen5-1forA, mamuIFITM3gen5-1rev, IF3gi-for (5’-GTGCCCACGTC AGTAGCTTTA-3’) or IF3gi-for2 (5’-TGTGCCCACGTCAGTAGCTTT-3’) and IF3gi-rev (5’-CTTCCTCACTCAGGCTCAGAC-3’) or IF3gi-rev2 (5’- CAAACTCTGAGCC AGCCAGC-3’). Amplicons were assembled on a scaffold sequence using Vector NTI ContigExpress and polymorphisms identified by visual inspection.

### Statistical analysis

AIDS-free survival analysis, including a log-rank test for *rhIFITM3* genotype-related differences, was performed using the LIFETEST procedure of the SAS software version 9.4 (SAS Institute Inc., Cary, NC). The association between *rhIFITM3* polymorphisms and virus load was assessed for statistical significance by means of either a Kruskal-Wallis test (three genotypes) or a Mann-Whitney test (two genotypes), as appropriate, using the GraphPad Prism software. All p-values were two-sided. The level of statistical significance was depicted as follows, *, p<0.05; **, p<0.01; ***, p<0.001.

## Results

### Rhesus macaque IFITM3 inhibits viral entry

**Fig 1:**
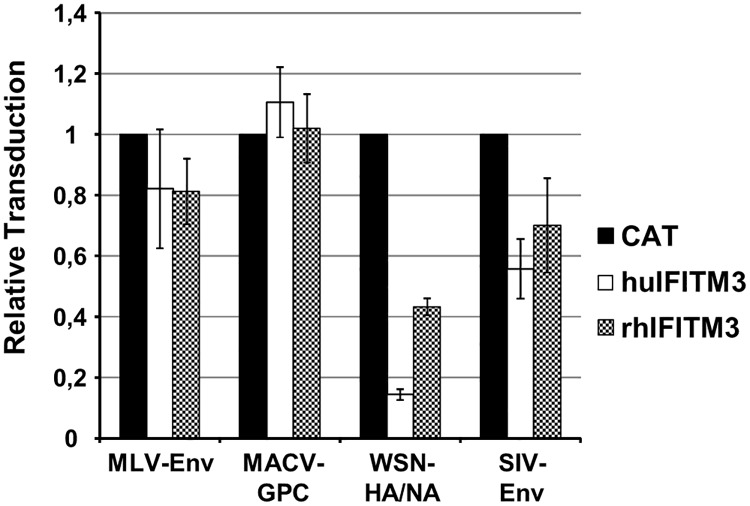
Rhesus macaque IFITM3 exerts antiviral activity. 293T cells expressing human (hu) and rhesus macaque (rh) IFITM3 or chloramphenicol-acetyltransferase (CAT) as negative control were transduced with MLV particles encoding luciferase and harboring the indicated viral glycoproteins. Luciferase activities in cell lysates were determined at 72 h post transduction. The average of three to six independent experiments is shown. Luciferase activities measured in control cells were set as 1. Error bars indicate standard error of the mean.

One *IFITM1* gene and two *IFITM3* genes have been identified in rhesus macaques. For the purpose of the present study, we will distinguish between the two *rhIFITM3* genes as *rhIFITM3* (LOC697829) and *rhIFITM3(2)* (LOC697564) because rh*IFITM3(2)* shows a slightly higher sequence homology to the human *IFITM2* gene than *rhIFITM3*. Here, we investigated whether rhIFITM3 inhibits viral entry in a way similar to human (hu) IFITM3, employing a pseudo-typing approach. Expression of huIFITM3 in 293T cells did not appreciably inhibit entry driven by the MLV envelope protein (Env) or the Machupo virus glycoprotein (MACV-GPC) whereas entry driven by the FLUAV-hemagglutinin (HA) was robustly inhibited (Fig 1), in keeping with published findings [4–6]. Entry driven by SIV-Env was also reduced upon huIFITM3 expression (Fig 1), again in agreement with published data [19, 20]. A similar tendency was observed for rhIFITM3 even though inhibition of SIV-Env- and FLUAV-HA-driven entry was less robust than in the case of huIFITM3 (Fig 1), despite comparable expression levels [6]. Thus, rhIFITM3 exerts antiviral activity but does so less efficiently than huIFITM3.

### Identification of polymorphisms in rhesus macaque *IFITM3*

**Fig 2:**
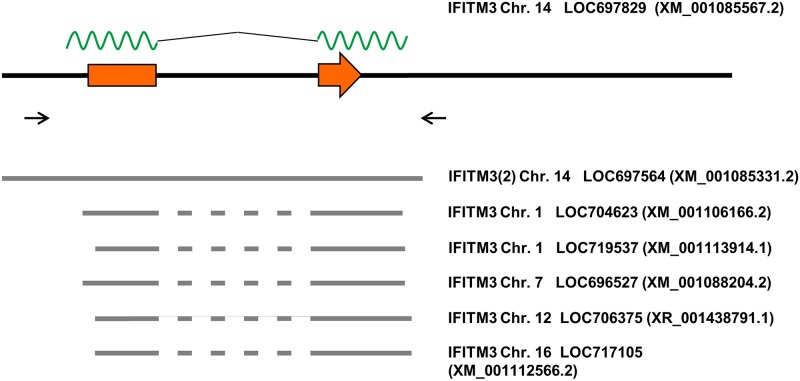
Overview of the *Macaca mulatta IFITM3* locus. The *Macaca mulatta IFITM3* locus is shown based upon assembly Mmul_051212 [41]. Exons are indicated as orange rectangles with an arrow giving the direction of translation. The mRNA is indicated above as a green wavy line, with the intron drawn as black line. The positions of the primers used for amplification are shown as arrows below the *IFITM3* locus. In the lower part, regions of homology to the highly similar *IFITM3(2)* gene and several pseudogenes are indicated. The missing introns in the pseudogenes are drawn as dashed lines. For each gene, the chromosome, gene symbol and reference accession number are indicated.

**Fig 3:**
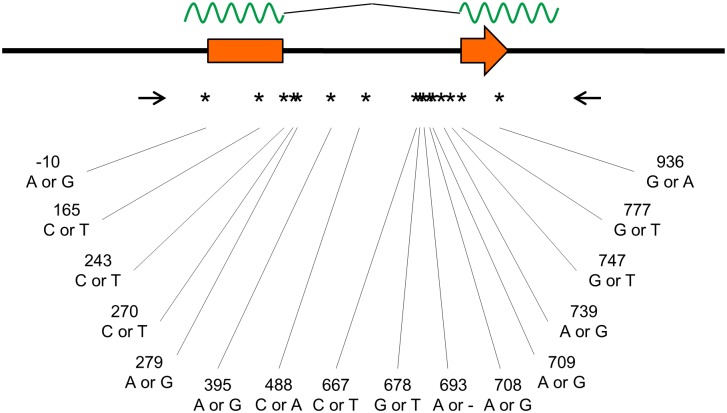
Overview of sequence variations identified in the *Macaca mulatta IFITM3* locus. The *Macaca mulatta IFITM3* locus and positions of primers are shown as in Fig 1. The positions of polymorphisms are marked by stars and the exact nucleotide position (relative to the start codon) and the detected variations are given.

We next aimed to characterize polymorphisms in the *rhIFITM3* gene based upon a previously characterized cohort of 39 SIV infected animals (cohort 1) (Table 1), where relationships between animals and confounding factors had been minimized [39]. To obtain results from a larger collection of animals, we randomly selected additional 55 animals for inclusion into a second cohort (cohort 2) (Table 1). The rhIFITM3 gene has two exons leading to a mature mRNA of 636 bp (XM_001085567.2) whereas the genomic region encoding the primary transcript comprises 1202 bp. We devised a PCR strategy to selectively amplify the genomic region of the *rhIFITM3* gene so as to yield a 1340 bp amplification product (Fig 2). The DNA sequence was determined directly from purified PCR products, and sequence variations were identified at 16 positions (Fig 3 and Table 2). Three polymorphisms were located in the coding region but did not change the amino acid sequence. Twelve polymorphisms (including one single nucleotide deletion) were intronic and one polymorphism was located upstream of the coding sequence. Seven polymorphisms had a low level of heterozygosity (≤10%) but, with few exceptions, the two cohorts were found to be characterized by similar major allele frequencies (MAF; Table 2). Polymorphism g.488 C>A was only detected in the second cohort. Polymorphisms g.279 G>A and g.678 G>T showed higher heterozygosity in cohort 2, while the opposite was true for g.693 delA, g.708 A>G, g.709 G>A and g.747 T>G. Finally, a polymorphism in the 5´region of the coding sequence that corresponds to rs12252 T>C in the human *IFITM3* gene was not identified in our sample.

**Table 2:** Frequency of polymorphisms.

*Position*	*Mutation*	*Cohort 1*		*Cohort 2*		*All animals*^a^	
		*Major Allele Frequency*	*Observed Heterozygosity*	*Major Allele Frequency*	*Observed Heterozygosity*	*Major Allele Frequency*	*Observed Heterozygosity*
g.-10 A>G	non-coding	0.95	0.10	0.96	0.07	0.96	0.08
c.165 T>C	coding, silent	0.68	0.49	0.79	0.38	0.75	0.42
c.243 C>T	coding, silent	0.95	0.10	0.96	0.08	0.96	0.08
g.270 C>T	non-coding	0.99	0.03	0.99	0.02	0.99	0.02
g.279 G>A	non-coding	0.94	0.08	0.89	0.22	0.91	0.16
g.395 A>G	non-coding	0.59	0.46	0.65	0.42	0.62	0.43
g.488 C>A	non-coding	1.00	1.00	0.95	0.11	0.97	0.06
g.667 C>T	non-coding	0.89	0.23	0.98	0.04	0.94	0.12
g.678 G>T	non-coding	0.96	0.08	0.88	0.24	0.92	0.17
g.693 delA	non-coding, deletion	0.71	0.39	0.90	0.20	0.81	0.27
g.708 A>G	non-coding	0.72	0.36	0.90	0.20	0.82	0.26
g.709 G>A	non-coding	0.72	0.36	0.90	0.20	0.82	0.26
g.739 G>A	non-coding	0.96	0.08	0.99	0.02	0.98	0.04
g.747 T>G	non-coding	0.65	0.54	0.78	0.36	0.73	0.44
g.777 G>T	non-coding	0.95	0.10	0.96	0.07	0.96	0.08
c.936 G>A	coding, silent	0.95	0.10	0.96	0.09	0.96	0.08

^a^**C**ohorts 1 and 2 plus one non-infected animal.

### Rhesus macaque *IFITM3* polymorphisms in SIV infection

**Fig 4:**
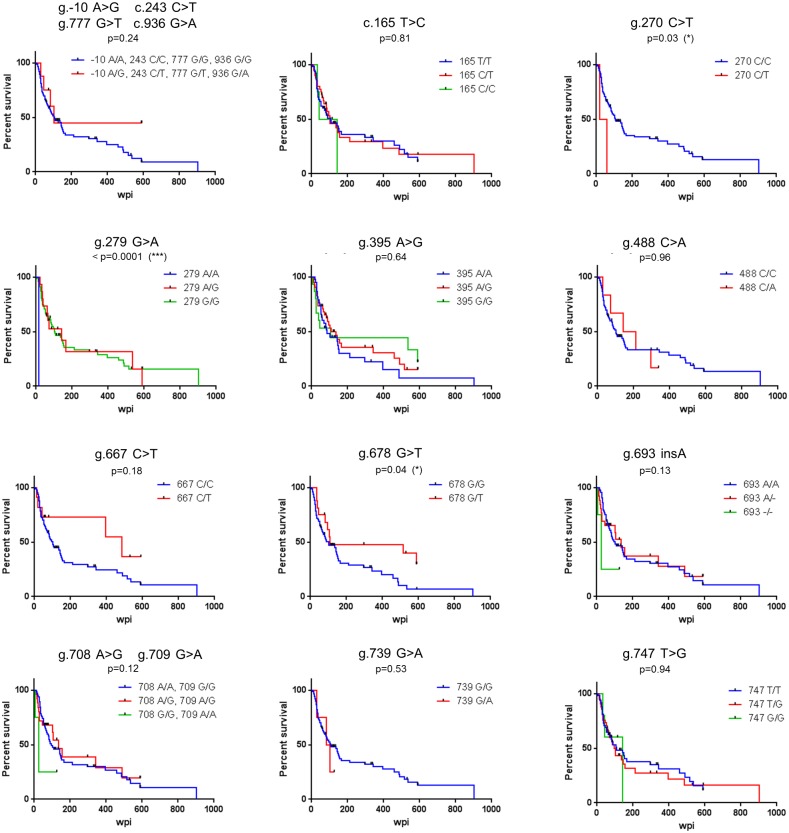
*IFITM3* alleles and disease progression in SIV-infected rhesus macaques. For each polymorphism, the AIDS free survival (percent survival) is shown in Kaplan-Meier plots. Censored animals are shown as black ticks. The polymorphisms are indicated above each diagram along with the p-values obtained by the log-rank test. AIDS free survival time is shown as weeks post infection (wpi). Statistical significant results are depicted as follows, *, p<0.05; **, p<0.01; ***, p<0.001.

To assess whether the detected sequence variations had an influence on the course of SIV infection, we first analyzed their effect on AIDS-free survival time (Fig 4, Table 3). Polymorphisms g.270 C>T (genotype GT) and g.678 G>T (genotype GG) showed a nominally significant association with faster disease progression when all animals from both cohorts were considered. However, these associations were not observed when each cohort was analyzed separately and were not significant when animals with MHC allele Mamu-A1*001 were excluded from the analysis (not shown). Polymorphism g.279 G>A showed a highly significant association of the AA genotype with faster disease progression (log-rank test < 0.0001) and this association was also detected when carriers of Mamu-A1*001 (log-rank test < 0.0001) were excluded (Fig 4, Table 3). However, this variant was only little polymorphic (MAF: 0.95), and the significance of the association hinged heavily on a single AA animal in cohort 1.

**Table 3:** Polymorphism-specific log-rank tests (p values) of genotype differences in AIDS-free survival.

*Position*	*Mutation*	*Cohort 1*	*Cohort 2*	*All animals*
g.-10 A>G	non-coding	0.8500	0.1923	0.2363
c.165 T>C	coding, silent	0.4035	0.6633	0.8121
c.243 C>T	coding, silent	0.8500	0.1923	0.2363
g.270 C>T	non-coding	0.0624	0.2469	(*) 0.0334
g.279 G>A	non-coding	(**) 0.0099	0.2505	(***) < 0.0001
g.395 A>G	non-coding	0.7883	0.3516	0.6444
g.488 C>A	non-coding	n.a.	0.9456	0.9636
g.667 C>T	non-coding	0.5739	0.1517	0.1828
g.678 G>T	non-coding	0.2668	0.1949	(*) 0.0437
g.693 delA	non-coding, deletion	0.2158	0.2295	0.1282
g.708 A>G	non-coding	0.2877	0.2295	0.1168
g.709 G>A	non-coding	0.2877	0.2295	0.1168
g.739 G>A	non-coding	0.8120	0.6230	0.5284
g.747 T>G	non-coding	0.7265	0.5876	0.9377
g.777 G>T	non-coding	0.8500	0.1923	0.2363
c.936 G>A	coding, silent	0.8500	0.1923	0.2363

Statistically significant results are depicted as follows,

*, p<0.05;

**, p<0.01;

***, p<0.001.

**n.a.** not applicable

**Fig 5:**
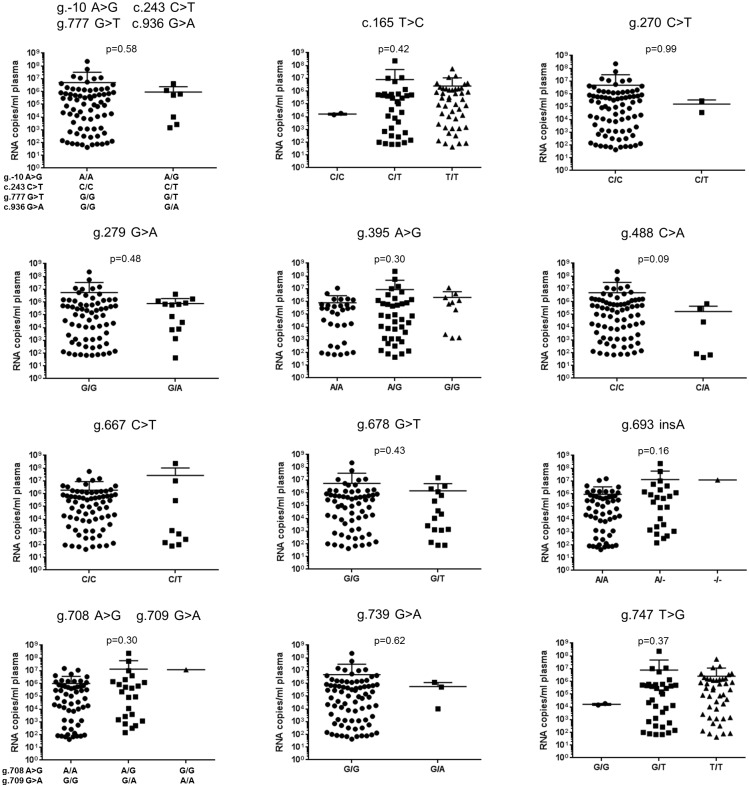
*IFITM3* alleles and viral load at set point in SIV-infected rhesus macaques. For each polymorphism, the virus load at set point (20 weeks post infection) was compared between the different genotypes. The polymorphisms are indicated above each diagram along with the p-values obtained by Mann-Whitney or Kruskal-Wallis test. Virus load is shown as RNA copies/mL plasma.

For a subset of 81 animals, virus load data were available that allowed the potential association between *rhIFITM3* polymorphism and SIV amplification to be analysed. However, no statistically significant association with plasma viral RNA copies at 20 weeks after infection or at time of death was noted for any of the polymorphisms (Fig 5, Table 4). Only in cohort 1, we found a nominally significant association of polymorphisms g.693 delA (genotype -/-), g.708 A>G (genotype GG) and g.709 G>A (genotype AA) with increased viral load and of polymorphism g.678 G>T (genotype GT) with lower virus load (Table 4). However, these findings were not replicated in cohort 2 and were not detected when the full set of animals was analyzed.

**Table 4:** Polymorphism-specific rank-based tests (p values) of genotype differences in virus load.

*Position*	*Mutation*	*Cohort 1*	*Cohort 2*	*All animals*
g.-10 A>G	non-coding	0.4850 ^a^	0.8086 ^a^	0.5841 ^a^
c.165 T>C	coding, silent	0.2760 ^a^	0.5211 ^a^	0.4229 ^b^
c.243 C>T	coding, silent	0.4850 ^a^	0.8086 ^a^	0.5841 ^a^
g.270 C>T	non-coding	n.a.	n.a.	0.9877 ^a^
g.279 G>A	non-coding	n.a.	0.7776 ^a^	0.4796 ^a^
g.395 A>G	non-coding	0.1233 ^b^	0.6677 ^b^	0.3022 ^b^
g.488 C>A	non-coding	n.a.	0.0715 ^a^	0.0923 ^a^
g.667 C>T	non-coding	0.2591 ^a^	0.5290 ^a^	0.1723 ^a^
g.678 G>T	non-coding	(*) 0.0227 ^a^	0.9722 ^a^	0.4257 ^a^
g.693 delA	non-coding, deletion	(**) 0.0078 ^a^	0.7628 ^a^	0.1628 ^a^
g.708 A>G	non-coding	(*) 0.0337 ^a^	0.7628 ^a^	0.2962 ^a^
g.709 G>A	non-coding	(*) 0.0337 ^a^	0.7628 ^a^	0.2962 ^a^
g.739 G>A	non-coding	0.8831 ^a^	n.a.	0.6158 ^a^
g.747 T>G	non-coding	0.2495 ^a^	0.5211 ^a^	0.3689 ^b^
g.777 G>T	non-coding	0.4850 ^a^	0.8086 ^a^	0.5841 ^a^
c.936 G>A	coding, silent	0.4850 ^a^	0.8086 ^a^	0.5841 ^a^

Statistically significant results are depicted as follows,

*, p<0.05;

**, p<0.01;

***, p<0.001.

^a^Mann-Whitney test (two genotypes present)

^b^Kruskal-Wallis test (three genotypes present)

**n.a.** not applicable

## Discussion

Immune-related IFITM proteins have been established as important antiviral effectors of the interferon response, and a polymorphism in the human *IFITM3* gene has been found to be associated with disease severity and progression in FLUAV and HIV-1 infection [27, 33]. Rhesus macaques are important animal models for human infections but it was hitherto unknown whether the *rhIFITM3* gene is polymorphic and whether possible polymorphisms would impact upon the course of SIV infection. We demonstrated that rhIFITM3 displays antiviral activity and identified 16 polymorphisms in *rhIFITM3*. Some of the polymorphisms were associated with either disease progression or viral load. However, none of the polymorphisms impacted on both parameters, and significant associations were not observed in cohort 2. Moreover, most associations failed to attain statistical significance when Mamu-A1*001 animals were excluded from the analysis, arguing against a major impact of *rhIFITM3* polymorphisms on SIV infection.

Recently, a comprehensive characterization of the antiviral activity of IFITM proteins of non-human primates revealed that these proteins, like their human counterparts, can block viral infection [20]. However, IFITM proteins from rhesus macaques were not included in these studies. Our results demonstrate that rhIFITM3 and huIFITM3 target the same viral glycoproteins, but with somewhat different efficiency. Thus, entry driven by FLUAV-HA and SIV-Env appears to be more efficiently inhibited by huIFITM3 than by rhIFITM3. These results are in agreement with our previous analysis demonstrating that huIFITM3 inhibits entry driven by filovirus glycoproteins with greater efficiency than rhIFITM3, despite comparable expression in cells [6]. In summary, rhIFITM3 inhibits entry of SIV in cell culture which raised the question whether *rhIFITM3* is polymorphic and whether possible polymorphisms would impact the course of SIV infection.

Our sequence analysis identified variations at 16 positions present in at least two out of 94 animals. Six of these polymorphisms were highly polymorphic (MAF <75%) and homozygous animals could be detected for both alleles. Three of the polymorphisms identified in our study were located within the coding sequences, but led to silent changes. This is noteworthy since the N-terminal residues of IFITM3 control intracellular localization and spectrum of antiviral activity [32, 43, 44] and were suggested to be shaped by selective pressure during primate evolution [32]. One polymorphism was located upstream of the coding sequence; all other polymorphisms were identified within the intron. We found no evidence for a polymorphism that would lead to the production of a truncated protein similar to rs12252-C in human *IFITM3*, a variant that is associated with the clinical severity of influenza disease [27]. Our results were in keeping with a previous report suggesting that *IFITM1* and *IFITM3* in non-human primates probably underwent purifying selection [20]. Thus, we did not find any non-synonymous polymorphisms, indicating that maintenance of the primary amino acid sequence is essential for the function of the protein in rhesus macaques.

Our analysis was based upon DNA sequences derived from assembly Mmul_051212 [41]. In the meantime, a new genomic framework (Mmul_8.0.1) has been published [45] where the sequences at the 3’ end of the coding regions of *rhIFITM3* (LOC697829) and *rhIFITM3(2)* (LOC697564) have been exchanged in comparison to the previous assembly (Mmul_051212). However, our previously reported cloning and sequencing of cDNAs from both loci [6] and the analysis presented here show better agreement with the *rhIFITM3* locus as annotated in the older genomic assembly. Therefore, we continued to use Mmul_051212 throughout this study. A reconstruction of haplotypes using PHASE (v. 2.1) [46] based upon our results revealed 16 haplotypes for the *rhIFITM3* gene, all of which clustered together and were clearly distinct from *rhIFITM3(2)* (data not shown). This reinsures us that all detected polymorphisms are true *rhIFITM3* polymorphisms.

Silent mutations may affect translational efficiency and protein folding [47] whereas polymorphisms in untranslated regions or introns may alter mRNA stability or splicing [48]. In consequence, we analyzed the association between the polymorphisms and survival time and viral load in SIV-infected rhesus macaques. For this, we used a sample that previously allowed detection of an association between *TLR7* polymorphisms and survival time (cohort 1, [39]) and a sample of randomly selected animals (cohort 2). When the full set of animals was considered, we identified three polymorphisms (g.270 C>T, g.279 G>A, g.678 G>T) with a nominally significant association between a genotype and AIDS-free survival, but not with virus load at set point (20 weeks post infection). However, for two of these polymorphisms the association was not significant if the two cohorts were analyzed separately or when animals with MHC allele Mamu-A1*001 were excluded from the analysis. The polymorphism g.279 G>A genotype AA showed a nominally significant association with AIDS progression even after adjustment for MHC alleles. However, this position is only modestly polymorphic and the association observed was dependent on a single animal with AA genotype. Collectively, these results suggest that *rhIFITM3* polymorphism might not play a major role in SIV infection.

In summary, our study not only demonstrated that rhIFITM3 displays antiviral activity, but also led to the identification of polymorphisms in the coding and non-coding region of the *IFITM3* gene of rhesus macaques. Notably, all polymorphism in the coding region were silent and strong evidence for an association of rhIFITM3 polymorphisms with disease progression and viral load in SIV infected animals was not obtained. We cannot exclude that the analysis of a substantially larger number of animals might reveal such associations. However, the frequency of potentially affected animals and/or the effect of the polymorphisms in terms of viral inhibition would be very low and are therefore not expected to significantly influence results obtained in the macaque model of AIDS.
